# Parental Punishment and Adolescents’ Loneliness: A Moderated Mediation Model of General Self-Concept and Teacher–Student Relationships

**DOI:** 10.3389/fpsyg.2021.693222

**Published:** 2021-11-02

**Authors:** Yun Luo, Anyi Wu, Hui Zhang

**Affiliations:** ^1^School of Education, Zhaoqing University, Zhaoqing, China; ^2^Jiangsu Key Laboratory of Mental Health and Cognitive Science, School of Psychology, Nanjing Normal University, Nanjing, China

**Keywords:** teenagers, loneliness, parental punishment, general self-concept, teacher–student relationships

## Abstract

**Background:** Loneliness adversely affects physical and mental health; therefore, it is necessary to explore its related influencing factors and mechanisms. This study investigated the mediating role of general self-concept in the association between parental punishment (PP) and adolescent loneliness and as well as the moderating role of teacher–student relationships (TSR) in Chinese students.

**Methods:** Data were obtained from 1,169 Chinese students (10–18years old) using several self-report questionnaires: the Egna Minnen av Barndoms Uppfostran (EMBU), Self-Description Questionnaire (SDQ), Teacher–Student Relationships Scale (TSR), and UCLA Loneliness Scale. Data were analyzed with IBM SPSS 22.0, and the PROCESS macro program.

**Results:** (1) Parental punishment had a positive predictive effect on adolescent loneliness, (2) parental punishment predicted adolescent loneliness not only directly but also indirectly through the mediating effect of general self-concept, and (3) teacher–student relationships moderated the influence of PP on adolescent loneliness.

**Conclusion:** Adolescent loneliness is less affected by parental punishment when TSRs are better. Additionally, when adolescents are punished less by their parents and have good teacher–student relationships, they have higher general self-concepts.

**Limitations:** This study’s cross-sectional research design was unable to show causal relationships among the factors influencing adolescent loneliness.

## Introduction

Adolescent loneliness is currently a topic of considerable interest among researchers. Loneliness refers to a distressing feeling experienced by individuals when their needs are unmet by their social networks (Cacioppo et al., 2015). Previous research has shown that loneliness occurs throughout life, but typically peaks during puberty (Qualter et al., 2015). When teenagers’ peers become more important in their life, parents tend to be positioned. Especially in the early adolescence, when young people are psychologically far away from their parents but have not found their place in the social world of the same age, some teenagers will feel lonely (Goossens, 2018). Loneliness is therefore a common negative emotional experience during adolescence (Shevlin et al., 2014; Goossens, 2018) that is detrimental to both physical and mental health (Holt-Lunstad et al., 2015; Kearns et al., 2015). Indeed, many studies have found that loneliness makes significantly impact to individuals and society, such as inducing depression (Kılınç et al., 2020; Wang et al., 2021), affecting social function (Tan et al., 2020), and leading to problematic behaviors (McKay et al., 2017). Overall, the above literature clearly shows that loneliness adversely affects physical and mental health. Therefore, it is necessary to explore the influencing factors and mechanisms of loneliness.

Parental punishment (PP) refers to circumstances in which parents purposefully and physically make children feel pain in order to correct or control their behaviour. Although such pain usually does not cause substantial physical harm, it may seriously affect mental health development during childhood (Straus and Kantor, 1994). Therefore, PP is an important factor affecting adolescent loneliness. Indeed, the parent-child relationship can cause a strong impact to teenagers, consequently, parental rearing patterns will affect their loneliness (Nayak and Kochar, 2016). Research has shown that strict parental discipline is positively correlated with behavioural problems among both children and adolescents and predicts the emergence of emotional problems (Danzig et al., 2015; Flouri and Midouhas, 2017; Pinquart, 2017). Early adolescents have some new characteristics in psychological and behavioural development, including increasing autonomy and new psychological needs for peer relationships (Shifflet-Chila et al., 2016). However, due to conflicts between the increased desire for autonomy and deep feelings of incompetence, adolescents still need parental guidance and support, the lack of which may lead to increased feelings of loneliness (Marcoen et al., 1987; Laursen and Hartl, 2013).

In adolescence, many factors may affect adolescents, including physiological factors related to puberty, cognitive ability related to more complexed thinking, identity development, changes in social roles, and changes in the environment for entering the working world. To be aware, in the late adolescence, teenagers began to stay away from family of origin (Shifflet-Chila et al., 2016). Self-development has become one of the important development tasks in this period (Crone and Fuligni, 2020). Self-concept refers to the perception and evaluation of oneself, which substantially influences psychological factors and behaviours (Bournelli et al., 2009; Guerin and Tatlow-Golden, 2019). According to the multilevel and multidimensional structural model for the self-concept (Shavelson et al., 1976), the two categories of self-concept are the general self-concept and domain-specific self-concepts. Clem et al. (2018) found that the concept of adolescents’ domain-specific self-concepts of ability can predict their domain-specific causal attribution. The general self-concept is positively correlated with job satisfaction, whereas it is negatively correlated with emotional exhaustion, depersonalization and reduced personal satisfaction (Nwafor et al., 2015). Self-concept is related to loneliness (Richman et al., 2016), which may cause students to gain fewer social skills and lower self-esteem (Wang et al., 2013).

According to theory of the looking-glass self of Cooley, individuals gradually form a “mirror self” through the “mirror process”; that is, individuals understand and define themselves based on other peoples’ attitudes, thereby forming corresponding self-concepts (Cooley, 1964). Mead further proposed that the “self” was generated through social experiences and activities and was the result of one’s relationships and how one is perceived by others; that is, key people in the social group significantly impact the formation of one’s self-concept (Mead, 1962). Indeed, many studies have found that good family relationships influence the adolescent self-concept (Lau and Leung, 1992; Mishna et al., 2016). Parents are vital in the lives of their children; their upbringing styles exert influence on the formation of the general self-concept during childhood (Chan and Koo, 2011). Previous studies have also shown that different parenting styles have an important impact on individual self-concept. For example, perceived parenting will affect children’s general self-concept (Chen et al., 2020). The parenting style based on supporting children helps create an atmosphere of influence and trust, which is conducive to the development of prosocial behavior and self-concept (Bagán et al., 2019). Coercive control from parents can lead to negative self-concept among adolescents (Boudreault-Bouchard et al., 2013).

To summarise, parental rearing patterns tend to leaving a strong affect on adolescents’ self-concept. Hence, this study speculated that parental punishment may directly or indirectly influent adolescents’ general self-concept, which then lead to the increase of loneliness. In addition, Huang et al. (2021) have found similar mechanisms which self-concept plays a mediating role in the relationship between parents-related factors and adolescents’ behavioral outcomes. Therefore, we hypothesize that the general self-concept mediates the influence of parental punishment on loneliness among middle-school students. An exploration of this mediating role may help answer the question of “how” parental punishment affects adolescent loneliness but cannot clarify when it becomes most significant. According to the theory of developmental situation, the essence of human development entails a dynamic and changing interaction between individuals and the diversified environment in which they live. The theory also emphasizes the systematic influences of various factors and their interactions on individual development (Lerner, 2001). As important actors in both the family and school environments, parents and teachers may interact to jointly influence student development (Greenwood and Hickman, 1991; Sawka et al., 2002) and behavioural adaptation (De Haan et al., 2014).

Students with good teacher–student relationships (TSR) have lower negative and higher positive senses of achievement (Lei et al., 2018). Conversely, poor TSR are associated with poorer student mental health, and students who feel they lack support from their teachers experience increased loneliness (Besevegis and Galanaki, 2010; Morin, 2020). It has also been shown that parental warmth and support for their adolescents can predict positive personality traits (Callina et al., 2014). Teacher-student relationships plays as a protective role in the process of the individual adaptive development from parent-child relationships (Sabol and Pianta, 2012). Parent-child attachment can significantly reduce children’s problematic behaviors, yet affected by a teacher-student relationships. To be noticed, when the teacher-student relationships is low, they are no longer related (Buyse et al., 2011). Therefore, good teacher–student relationships can help maintain healthy self-concept levels while reducing loneliness among teenagers, even when parents adopt negative upbringing methods (e.g., punishment and severity).

**Figure 1:**
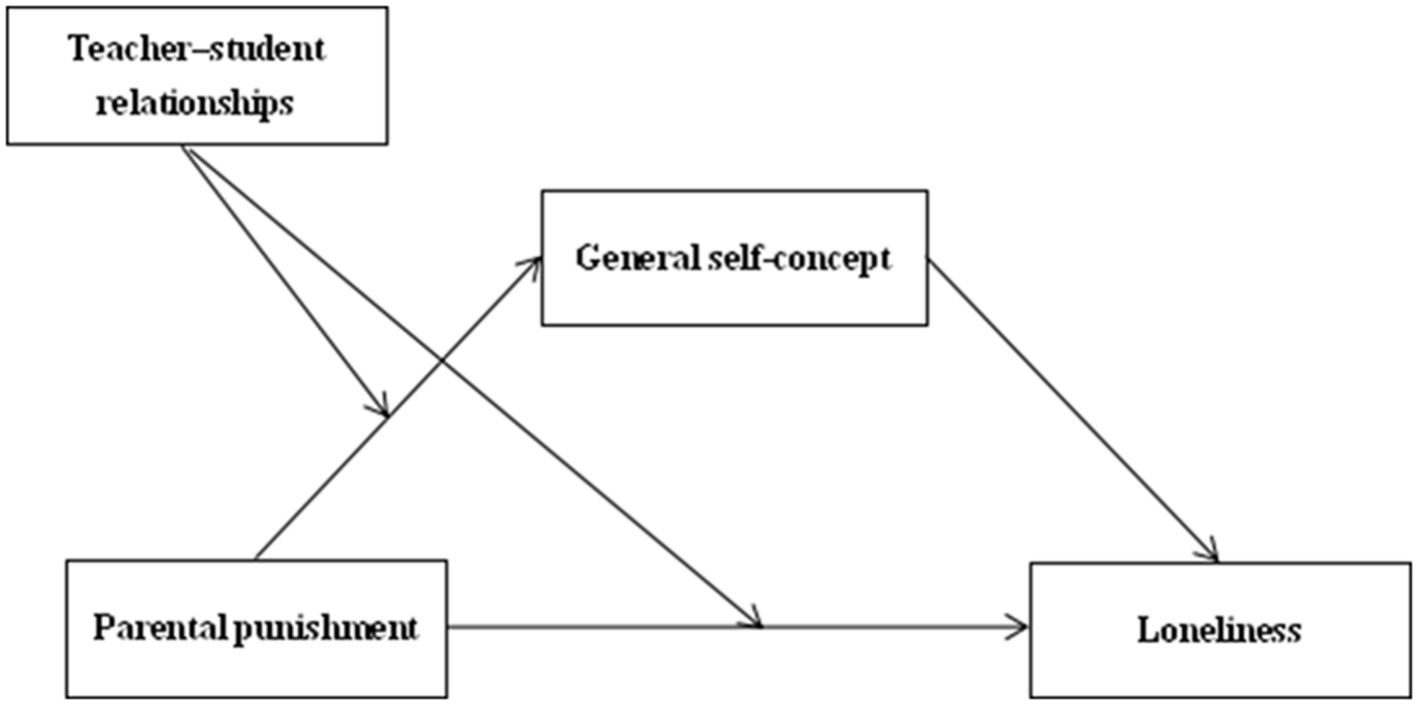
Hypothetical model.

In sum, a moderated mediation model can be constructed based on the mirror-self theory and developmental situation theory. Figure 1 is a hypothetical model of the mediating effect of general self-concept and the moderating effect of teacher–student relationships. In this model, parental punishment affects adolescent loneliness through the mediating role of general self-concept, while the teacher–student relationships regulates the first half of the path and the direct path, thus answering the question of “how” and “under what circumstances” parental punishment affects adolescent loneliness.

## Materials and Methods

### Participants and Data Collection

Participants were 1,169 students who were recruited from schools in Shaanxi Province, China, with an age range from 10 to 18years (*M*=13.24, *SD*=1.28). The sample included 600 boys and 569 girls (890 junior and 279 senior-high-school students). Each participant was asked to complete a total of four questionnaires, which are described in the following subsections.

### Measures

#### Parental Punishment

This study assessed parental punishment using the Parental Punishment Severity Subscale of Revised Parenting Style Evaluation Scale (i.e., the Egna Minnen av Barndoms Uppfostran; EMBU; Yue et al., 1993). The original EMBU scale was developed to retrospectively measure the respondent’s perception and experience of their parents’ parenting behaviour. The scale includes four core dimensions: rejection, emotional warmth, overprotection, and favouring subjects, i.e., favouritism. Yue et al. (1993) analyzed all 81 items of the original scale and extracted six main factors from the father’s parenting style and five main factors from the mother’s parenting style. The revised scale includes the main variable of interest in this study: parental punishment. Specifically, the father’s scale includes six dimensions: understanding of emotional warmth, severe punishment, excessive interference, preference for subjects, rejection and denial, and overprotection. The mother’s scale includes five dimensions: emotional warmth and understanding, over-intervention and overprotection, rejection and denial, severe punishment, and preference. The severe punishment subscale comprises 11 items for fathers, which were developed out of 12 items from the original scale. Upon investigation, item 49 of the father severe punishment subscale, “if anything happens, I am often the only one who is blamed among brothers and sisters,” was removed because this question cannot be answered if the respondents are only children. Nine items were used for mothers, and all items were scored on a four-point scale ranging from “Never” to “Always.” The items included statements such as the following: “Even for minor mistakes, my parents punish me.” The scale measures respondents’ perception of what they recalled about both their father’s and mother’s punishment methods. Parents’ punishment scores were obtained by calculating from the average of mother’s and father’s punishment scores. Final scores indicated overall parental punishment severity; higher scores indicated more severe perceptions of parental punishment. This study’s internal consistency coefficients were 0.88 and 0.89 for the father’s and mother’s punishment severity factors, respectively. The internal consistency coefficient of the full subscale was 0.93.

#### General Self-Concept

General self-concept was measured using a subscale in the Self-Description Questionnaire (SDQ; Marsh et al., 1983, 1984). The Chinese version of the Self-Description Questionnaire II (SDQII) was revised by Chen and Cui (1997) and has acceptable reliability and validity. Items were developed based on the theory of the general self-concept proposed by Shavelson et al. (1976). Specifically, 10 items (e.g., “I am often relatively relaxed”) were answered using a six-point scale ranging from “completely consistent” to “completely inconsistent.” Reverse scoring was conducted to facilitate the data analytic process. Average scores for the subscale were then calculated, with higher scores indicating better general self-concepts. In this study, the scale had an internal consistency coefficient rating of 0.84.

#### Teacher–Student Relationships

The teacher–student relationships was measured using the teacher–student relationships scale compiled by Chu (2006), which consists of 18 questions that are divided into three factors (i.e., the learning relationship between teachers and students, the emotional relationship between teachers and students, and the status relationship between teachers and students). Selected items were scored so that 1 point was given for each “Yes” answer, while −1 point was given for each “No” answer (e.g., “The teacher is a little indifferent to me”). Reverse scoring was conducted to facilitate the data analytic process. Average scores were then calculated, with higher scores indicating better teacher–student relationships. In this study, the scale had an internal consistency coefficient rating of 0.87.

#### UCLA Loneliness Scale

The UCLA Loneliness Scale was developed by Russell (1996). We used the Chinese version revised by Wang et al. (1999). The scale was developed to measure loneliness caused by “the gap between the desire for social interaction and the actual level” while focusing on individual subjective experiences. It consists of 20 items that are scored on a four-point scale ranging from “never felt this way” to “always felt this way.” Of these items, nine were scored in reverse order. The overriding topic was “Do you often feel that you are not close to anyone?” Higher scores indicated higher levels of loneliness. The scale had an internal consistency coefficient of 0.83 in this study.

### Research Procedures and Statistical Analysis

Respondents completed all questionnaires in the same sitting. The IBM SPSS 22.0 software was used to analyze the resulting data, and the PROCESS macro program was used for testing the moderated mediating effect. The moderated mediation model is a model that contains both mediation and moderation variables. In this model, independent variables influence dependent variables through mediation variables, while the mediation process is moderated by moderation variables (Baron and Kenny, 1986; Wen and Ye, 2014). In our study, the independent variable was parental punishment, the dependent variable was loneliness, the mediation variable was general self-concept, and the moderation variable was teacher–student relationships. The moderated mediating effect included both the partial mediating effect of general self-concept on the relationship between parental punishment and adolescent loneliness and the moderating effect of the teacher–student relationships between parental punishment and adolescent loneliness. The Bootstrap method was used to test the significance of the regression coefficients. The sample distribution was reconstructed *via* random sampling with return. A total of 5,000 samples were constructed in this study; each sample size was 1,169, and we calculated the SE and CI of parameter estimation. Results were considered statistically significant when the CI did not include zero.

## Results

### Check for Common Method Bias

The study data was from students’ self-reports, so there may have been a common method bias. Based on the investigation of confidentiality and the reverse scoring of some items, a Harman single factor test was used to test data for common method bias (Podsakoff and Organ, 1986). The results showed that 13 factors with a characteristic root greater than 1 were obtained without rotation, and the variance revealed by the first factor was 19.03% (<40%). Therefore, the results indicated there was no serious common method bias in this study.

### Descriptive Results

Table 1 shows the average values, SDs, and correlation matrices for each variable. Loneliness was positively correlated with parental punishment (*p*<0.001) and negatively correlated with general self-concept and the teacher–student relationships (*p*<0.001). Further, parental punishment was negatively correlated with general self-concept and the teacher–student relationships (*p*<0.001). Finally, general self-concept was positively correlated with the teacher–student relationships (*p*<0.001).

**Table 1:** Descriptive statistics and correlation coefficients of the key study variables (*n*=1, 169).

Variable	1	2	3	4
1. Loneliness	_			
2. Parental punishment	0.25^***^	_		
3. General self-concept	−0.54^***^	−0.28^***^	_	
4. Teacher-student relationships	−0.36^***^	−0.32^***^	0.34^***^	_
*M*	2.08	1.57	4.50	0.30
*SD*	0.47	0.52	0.81	0.51

*** *p<0.001*.

### Moderated Mediation Model Test With Adjustment

The mediation effect analysis program (Zhao et al., 2010) as implemented in Model 8 of the SPSS macro program PROCESS v3.0 (Hayes, 2013) was used to test the direct path and first-half path of the moderated mediation model. First, all variables were standardized. As shown in Table 2, results indicated that parental punishment significantly and positively predicted loneliness. Here, the total effect was also significant. Furthermore, the direct predictive effect of parental punishment on loneliness remained significant after adding the mediator and moderator variables. At the same time, the negative predictive effect of parental punishment on general self-concept was significant, while the negative predictive effect of general self-concept on loneliness was also significant, thus indicating that general self-concept partially mediated the influence of parental punishment on loneliness (relative effect value of 55.40%). In addition, the interaction between parental punishment and teacher–student relationships had a significant predictive effect on general self-concept (*β*=−0.16, *t*=−2.26, *p*<0.05), thus indicating that the teacher–student relationships moderated the influence of parental punishment on general self-concept. Specifically, the teacher–student relationships regulated the first half of the mediating model, while the interaction between parental punishment and the teacher–student relationships played a significant role in predicting loneliness (*β*=0.13, *t*=2.99, *p*<0.01). This finding indicates that the teacher–student relationships plays a moderating role in the direct path of parental punishment to loneliness; as such, the teacher–student relationships regulates the direct path of parental punishment to loneliness and the first-half path of the intermediary model.

**Table 2:** The moderated mediation effect test of parental punishment (PP) on loneliness (*n*=1, 169).

Predictors	Loneliness			General self-concept			Loneliness		
	*β*	*t*	95%CI	*β*	*t*	95%CI	*β*	*t*	95%CI
Parental punishment	0.25	8.96^***^	[0.18, 0.28]	−0.32	−6.97^***^	[−0.40, −0.23]	0 0.08	3.05^**^	[0.03, 0.13]
Teacher-student relationships (TSR)				0.45	10.13^***^	[0.36, 0.54]	−0.18	−6.76^***^	[−0.23, −0.13]
PP×TSR				−0.16	−2.26^*^	[−0.30, −0.02]	0 0.13	2.99^**^	[0.04, 0.21]
General self-concept							−0.26	−16.09^***^	[−0.30, −0.23]
*R* ^2^	0.06			0.15			0.34		
*F*	80.24^***^			74.16^***^			161.91^***^		

*β=standardized coefficients*.

* *p<0.05*;

** *p<0.01*;

*** *p<0.001*.

**Figure 2:**
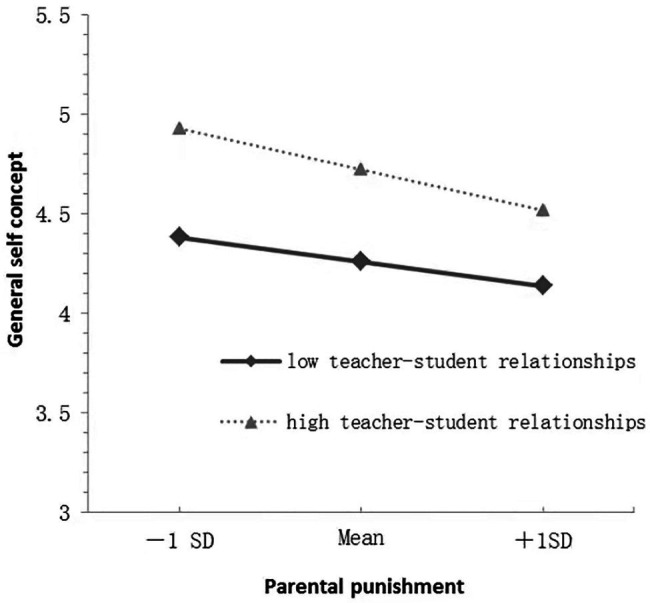
Plot of the interaction between parental punishment and teacher-student relationships on general self-concept.

**Figure 3:**
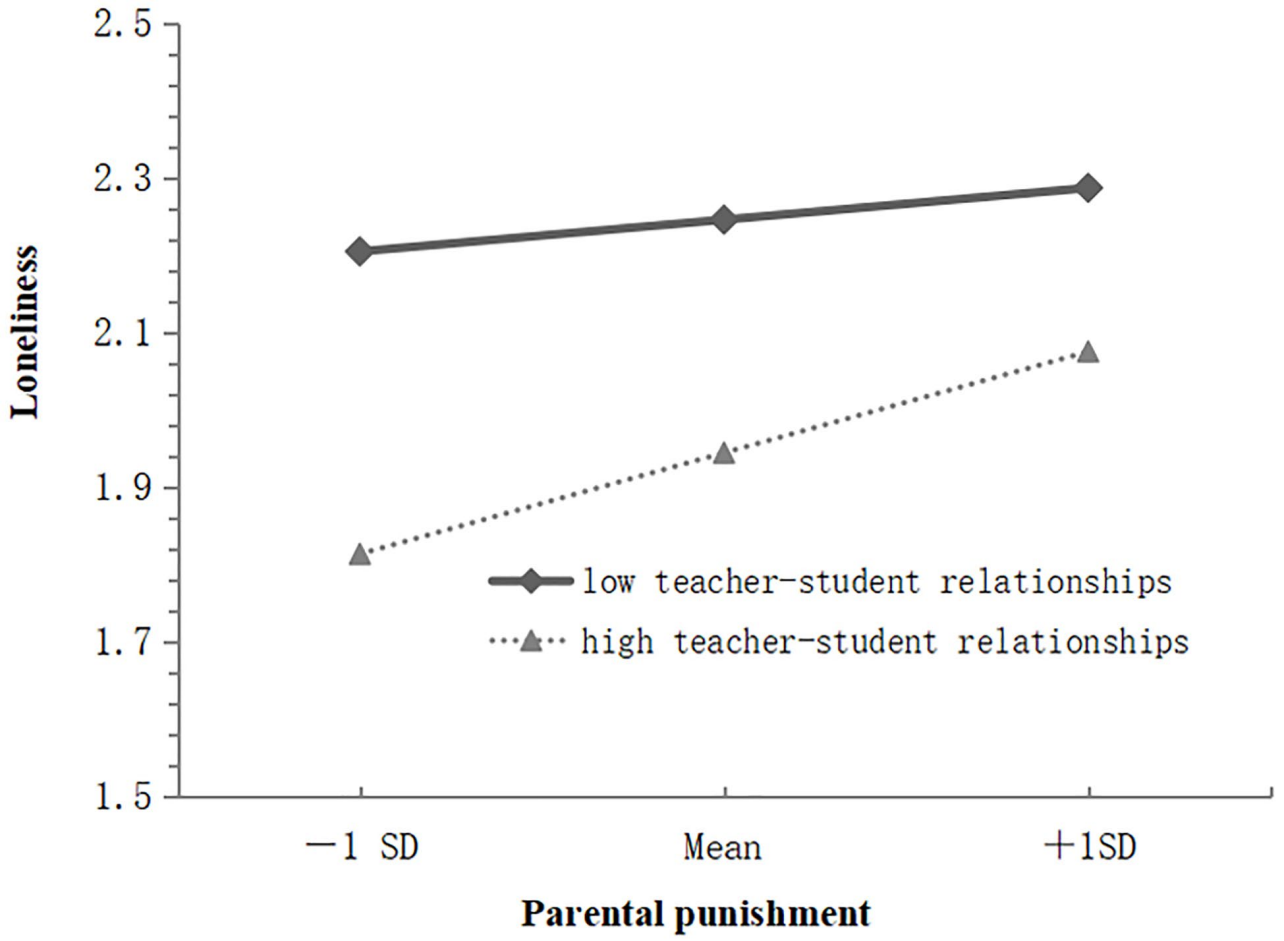
Plot of the interaction between parental punishment and teacher-student relationships on loneliness.

The moderating effect of the student relationships was analyzed *via* a simple slope analysis graph to reveal the essence of the interaction (Dearing and Hamilton, 2006). In this context, the moderating variables were grouped by both positive and negative one SDs of the average; the average plus one SD was the high teacher–student relationships group, while the average minus one SD was the low teacher–student relationships group. In Figure 2, the results for the high teacher–student relationships group show that parental punishment negatively predicted general self-concept (*simple slope*=−0.40, *t*=−6.37, *p*<0.001) and parental punishment predicted general self-concept for participants in the low teacher–student relationships group (*simple slope*=−0.23, *t*=−4.43, *p*<0.001). In Figure 3, the results for the high teacher–student relationships group show that parental punishment positively predicted loneliness (*simple slope*=0.25, *t*=6.02, *p*<0.001) and parental punishment predicted loneliness for participants in the low teacher–student relationships group (*simple slope*=0.08, *t*=2.65, *p*<0.01).

## Discussion

This study investigated the mediating role of general self-concept in the association between parental punishment and adolescent loneliness and the moderating role of teacher–student relationships among Chinese students. The factor of general self-concept partially mediated the relationships between parental punishment and adolescent loneliness, which was consistent with our theoretical hypothesis. That is, we posited that parental punishment would predict adolescent loneliness through a direct pathway but could also be mediated by general self-concept. This result is congruent with previous findings regarding the influence of parental upbringing styles on both the general self-concept. For example, research shows that if parents adopt positive parenting methods, such as parental support, it will help teenagers to form positive development results, such as prosocial behavior and self-concept development (Bagán et al., 2019). However, negative parenting styles in which adolescents experience parental punishment, parental rejection, and low parental emotional warmth lead to negative developmental outcomes for adolescents, including increased aggressive behaviour and a negative general self-concept (Wu et al., 2015; Zubizarreta et al., 2019; Luo et al., 2020). Poor self-concept will lead to negative emotional experience, and it is easy to experience loneliness (Richman et al., 2016). Attachment theory suggests that an individual’s parent–child attachment experiences become internalized and generalized into internal working models about the self and/or others (Bretherton and Munholland, 1999; Pallini et al., 2014). The internal working model of the self includes two components – self-representation and others’ representation – that interact to form the individual’s attachment type. Specifically, higher-level parent–child attachments are likely to occur when parents use positive upbringing styles (e.g., caring, understanding, and support). Under these conditions, children are more likely to form positive psychological representations of their parents, thereby achieving higher self-esteem and self-worth (Bowlby, 1982). As a result, children develop positive self-beliefs through which they feel happier and more valuable. Conversely, lower-level parent–child attachments form when parents adopt negative upbringing styles (e.g., indifference, rejection, and rudeness), which may damage children’s sense of self-identity and value (Drake and Ginsburg, 2012). Development is influenced by the attachment type, as self-beliefs are first reflected through individual interpersonal relationships that then become generalized, thus affecting self-beliefs in other domains (Bowlby, 1980). Ultimately, they impact self-esteem, self-efficacy, and the self-concept, thereby influencing one’s level of loneliness (Al-Yagon and Mikulincer, 2004).

In Chinese traditional culture, it is generally believed that parents or other caregivers have the right to discipline children as they see fit. It is often said that “A child cannot do anything without fighting,” “A filial son comes out under a stick,” “If a son does not teach, it’s the father’s fault,” and “If jade is not polished, it is not made into a weapon,” and it is believed that corporal punishment is the socialization of children by parents in a socially acceptable way (Yu and Chen, 2011). Parents strictly discipline their children and believe they must educate their children by beating and scolding them. Therefore, some parents inevitably use more severe punishment than others to educate their children. Studies have shown that Chinese parents have more control over their children and are more authoritarian, while Western parents are more affectionate and receptive to their children (Chen et al., 1998); that is, Chinese parents’ parenting style is more severe or punitive than that of Western parents. However, research findings on Chinese students indicate that when parents are warm and emotionally supportive of their children, their children have positive self-evaluations. Conversely, parents’ rejection or denial of their children, severe punishment, and excessive protection cause children to constantly experience their incompetence and failure (Wang and Liu, 2005). If children often experience criticism, humiliation, and punishment, they cannot feel their parents’ love and care and are relatively alienated from an emotional connection with their parents. They also cannot establish a close relationship with their parents and tend to feel lonely.

This study examined the mediating role of general self-concept between parental punishment and loneliness and further explored the moderating role of teacher–student relationships in this mediating process. Results showed that teacher–student relationships regulate both the direct path and first-half path of the intermediary process, which suggests that the teacher–student relationships enhances the predictive effect of parental punishment on the general self-concept and loneliness among adolescents. Results also support previous findings that the teacher–student relationships plays a critical role in protecting healthy growth and development (Lei et al., 2018; Guirong et al., 2019; Engels et al., 2021). The teacher–student relationships regulated the direct path of parental punishment to predict loneliness; that is, parental punishment positively predicted adolescent loneliness when the teacher–student relationships was high. This is likely because teachers tend to provide more support and help to their students in the context of such a relationship, which encourages adolescents to receive help from others, including their parents (Ryan et al., 1994), which in turn increases positive emotional experiences while reducing feelings of loneliness. The teacher–student relationships also regulated the predictive effect of parental punishment on adolescent general self-concept. Unlike adolescents who have poor teacher–student relationships, those with good relationships experience more significant mediating effects from the general self-concept. Indeed, according to the developmental situation theory, parents and teachers (as important adults in the lives of children) may interact in ways that jointly influence areas of adolescent development (Greenwood and Hickman, 1991; Sawka et al., 2002) and behavioural adaptation (De Haan et al., 2014).

Based on the internal working model of attachment theory, individuals with higher-level parent–child attachments are more likely to form positive psychological representations of their parents while having higher self-esteem and self-worth (Bowlby, 1982). When accompanied by good teacher–student relationships, adolescents can develop more positive self-concepts and more properly engage in emotional management (Bergin and Bergin, 2009). In short, the teacher–student relationships is an important mediator of the predictive association between parental punishment and the adolescent general self-concept. Teacher–student relationships may significantly impact adolescents, particularly in the Chinese culture. Traditionally, the Chinese regard the relationship between teachers and students as a “blood relationship” or a family relationship of “1 day as a teacher and lifelong as a father,” which is an idea that is conducive to improving the intimacy of teacher–student relationships (Yao, 2010). Therefore, with good teacher–student relationships, adolescents can experience a sense of intimacy that naturally alleviates their parents’ influence.

Further, the moderating effect of the teacher–student relationships suggests that educators should pay more attention to its cultivation, especially because it is a vertical and unequal interpersonal relationship in which teachers are mainly expected to provide social support only in academics (Vandell and Dempsey, 1991). Due to this limited influence, teachers should also work to strengthen communication and cooperation with parents, thus helping students solve interpersonal and other psychological problems while reducing their experiences with loneliness.

### Limitations and Suggestions

This study had some limitations; below, we have provided suggestions for addressing them in future research. First, this study adopted a cross-sectional research design to investigate the factors influencing adolescent loneliness but was therefore unable to draw causal inferences. This limitation may have distorted the proportions of the mediating effects (Maxwell and Cole, 2007). Future studies should employ novel experimental research approaches or adopt longitudinal designs to clarify the causal relationships between variables. Second, this study used convenience sampling to recruit participants from a specific area. As such, caution should be taken when generalizing the results. Future studies may consider using a larger sample size to increase sample representativeness, thus improving the external validity of any conclusions. Third, this study only examined perceived teacher–student relationships among participants, and therefore did not investigate specific relationship types (e.g., intimacy, conflict, and attachment). Therefore, it is difficult to obtain a full picture of the interactions between parental upbringing systems and the teacher–student relationships. Fourth, the difference in parenting styles between the mother and father could be not addressed. For example, in the general Asian culture, mothers tend to have more responsibilities at home. However, mothers and fathers vary in other dimensions; some fathers might be full of emotional warmth, while some mothers may be punitive. Therefore, the question of how parenting styles vary by gender is a question for further research.

This study brings a new perspective to research targeted at reducing loneliness among adolescents and highlights key areas of practical significance. First, we should pay careful attention to the parental upbringing styles when attempting to prevent episodes of loneliness. As suggested by previous research, parenting styles have substantial effects on the general self-concept as well as behavioural outcomes (Bagán et al., 2019; Chen et al., 2020). Parents should thus reduce the use of negative educational methods in order to meet the emotional needs of their children within the family context while also paying more attention to their formative processes and levels of loneliness. Second, parents should actively facilitate the development of the general self-concept in their adolescents, which can effectively curb the adverse effects of parental punishment on adolescent loneliness. Parents and teachers should work to help adolescents gain awareness of themselves and their environment through understanding and respect while guiding them in the formation of positive general self-concepts. Third, more attention should be paid to developing the teacher–student relationships, which affects essential aspects of adolescent mental health (e.g., the sense of accomplishment, school engagement, and self-esteem; Lei et al., 2018; Guirong et al., 2019; Engels et al., 2021). A good teacher–student relationships can effectively promote the general self-concept in addition to preventing loneliness among teenagers. Teachers can strengthen communication and cooperation with parents in order to support their adolescents’ development. Finally, apart from influence of parents and teachers in adolescents’ loneliness, peers are also very important to their mental health. Therefore, in the future research, relationship with friends or classmates might be involved as well.

## Conclusion

In conclusion, parental punishment may not only directly predict adolescent loneliness but can also indirectly affect adolescent loneliness through the general self-concept. The teacher–student relationships also moderates the influence of parental punishment on the adolescent general self-concept; adolescent loneliness is less affected by parental punishment when the teacher–student relationships is better. However, when adolescents are punished less by their parents and have a good teacher–student relationships, they have a higher general self-concept. In addition, a good teacher–student relationships can help adolescents have a higher general self-concept and reduce their levels of loneliness.

## Data Availability Statement

The raw data supporting the conclusions of this article will be made available by the authors, without undue reservation.

## Ethics Statement

The studies involving human participants were reviewed and approved by Institutional Review Board at Nanjing Normal University. Written informed consent to participate in this study was provided by the participants’ legal guardian/next of kin.

## Author Contributions

YL: conceptualization, methodology, investigation, formal analysis, writing-original draft, and writing-reviewing and editing. AW: formal analysis and writing-original draft. HZ: data curation and writing-reviewing and editing. All authors contributed to the article and approved the submitted version.

## Funding

This study was funded by the general topic of pedagogy during the 13^th^ Five-Year planning of National Social Science Foundation “Research on the Present Situation, Influencing Mechanism and Coping Strategies of Young Students’ Academic Frustration Based on Core Literacy” (No. BBA180078).

## Conflict of Interest

The authors declare that the research was conducted in the absence of any commercial or financial relationships that could be construed as a potential conflict of interest.

## Publisher’s Note

All claims expressed in this article are solely those of the authors and do not necessarily represent those of their affiliated organizations, or those of the publisher, the editors and the reviewers. Any product that may be evaluated in this article, or claim that may be made by its manufacturer, is not guaranteed or endorsed by the publisher.
